# RIM Mutants Occlude the Effects of Aliphatic Alcohols on *C. elegans* Locomotion

**DOI:** 10.17912/micropub.biology.001611

**Published:** 2025-06-12

**Authors:** Natalie J. Guzikowski, Casey D. Gailey, David M. Miller III, Ege T. Kavalali

**Affiliations:** 1 Pharmacology, Vanderbilt University, Nashville, Tennessee, United States; 2 Cell and Developmental Biology, Vanderbilt University, Nashville, Tennessee, United States

## Abstract

Biomolecular condensates formed by liquid-liquid phase separation (LLPS) are emerging as a critical organizing principle for synaptic compartmentalization. Aliphatic alcohols are commonly used to disrupt LLPS in biochemical studies and in cells. Here, we tested the impact of aliphatic alcohols at the organismal level in *C. elegans* . We observe that aliphatic alcohols impair swimming behavior in wild-type controls but not in RIM/ UNC-10 mutant strains. Thus, our results suggest an *in vivo* correlate to the previously reported role of RIM liquid condensates in synaptic function in reduced systems.

**Figure 1. The effect of aliphatic alcohols on C. elegans swimming locomotion:**
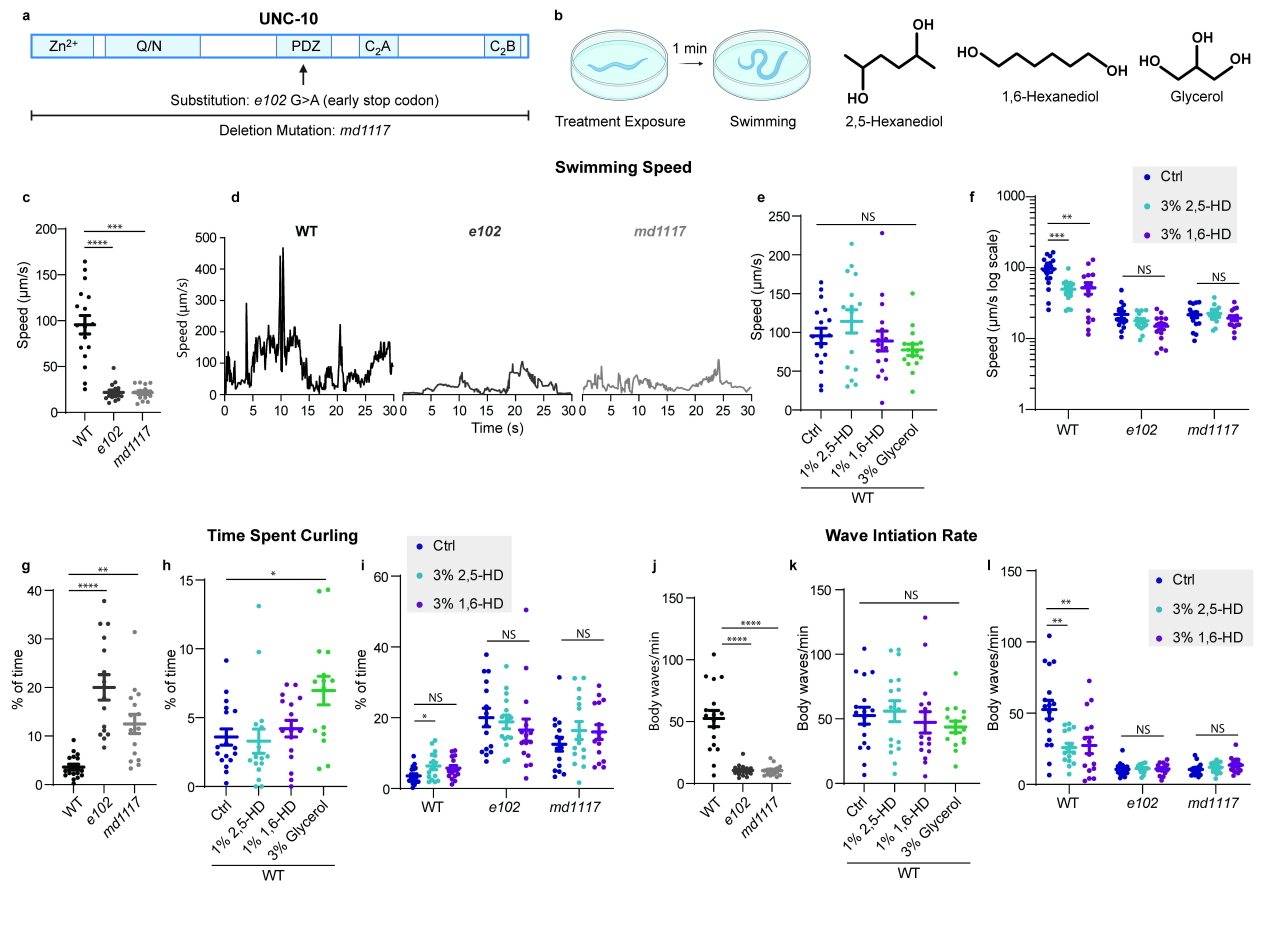
**a.** UNC-10 protein domain organization denoting mutations used in this study. **b.** Schematic of swimming assay and chemical structures of aliphatic alcohols. **c.** Comparison of average swimming speed between the WT (wild-type) and *unc-10* mutants **d.** Example traces of an individual trial from each genotype. **e.** Quantification of effects of 1% 2,5-HD, 1% 1,6-HD, and 3% glycerol on WT average swimming speed vs no-treatment control (Ctrl) and **f.** 3% 2,5-HD and 3% 1,6-HD on average swimming across the three genotypes. **g.** Comparison of the percentage of time spent curling during the swimming assay between the three genotypes. **h.** Quantification of 1% 2,5-HD, 1% 1,6-HD, and 3% glycerol on WT percentage of time spent curling and of **i.** 3% 2,5-HD and 3% 1,6-HD on percentage of time spent curling across the three genotypes. **j.** Comparison of the wave initiation rate during the swimming assay between the three genotypes. **k.** Quantification of 1% 2,5-HD, 1% 1,6-HD, and 3% glycerol on WT wave initiation rate and of **l.** 3% 2,5-HD and 3% 1,6-HD on wave initiation rate across the three genotypes. Values are mean ± SEM. Significance reported as *p < 0.05, **p < 0.01, ***p < 0.001, and ****p < 0.0001. WT Ctrl = 17, WT 1% 2,5-HD = 16, WT 1% 1,6-HD = 16, WT 3% glycerol = 15, WT 3% 2,5-HD = 15, WT 3% 1,6-HD = 15, *e102* Ctrl = 15, *e102* 3% 2,5-HD = 15, *e102* 3% 1,6-HD = 15, *md1117* Ctrl = 15, *md1117* 3% 2,5-HD = 15, *md1117* 3% 1,6-HD = 15.

## Description

Liquid-liquid phase separation (LLPS) is increasingly recognized as a fundamental mechanism for spatial organization within neurons and particularly at synapses. By partitioning biomolecules into dynamic, membrane-less compartments, LLPS enables the segregation of protein assemblies, creating distinct units that are otherwise fluid with the cytosol. Protein interactions governed by both structured domains and LLPS-defined interactions, mediate subcellular organization and neuronal physiology. Despite a of myriad data demonstrating that synaptic proteins phase separate, less is known of the specific functional roles of liquid condensates in synaptic function and behavior (Milovanovic et al., 2018; Zeng et al., 2018, 2019; Wu et al., 2019, 2023; Bai et al., 2021; Day et al., 2021; Emperador-Melero et al., 2021; Park et al., 2021, 2023; Imoto et al., 2022; Zhu et al., 2024; Zhang et al., 2025).

The pre-synaptic scaffold protein RIM (Rab3 Interacting Molecule) is fundamental to synaptic signaling. The phase separation of RIM with RIM-BP (RIM Binding Protein) effectively cluster VGCCs (Voltage Gated Calcium Channels) at the membrane (Wu et al., 2019). Interestingly, when neuronal phase separation is disrupted with the aliphatic alcohols, RIM nanoclustering and consequent evoked neurotransmission efficacy are impaired whereas spontaneous release is preserved. Remarkably, treatment with aliphatic alcohols does not disrupt the residual evoked release in RIM1 loss-of-function mutants (Guzikowski and Kavalali, 2024). These findings indicate that LLPS functionally organizes protein assemblies at nanoscale, enabling the precise regulation of distinct modes of neurotransmission (i.e., evoked vs spontaneous).

To explore a behavioral correlate of RIM-dependent LLPS at the synapse, we utilized the nematode *Caenorhabditis elegans* . The *C. elegans* RIM homolog, UNC-10 , was originally identified as an uncoordinated mutant (Brenner, 1974). The core functions of RIM are conserved between vertebrates and nematodes with key roles for both UNC-10 and RIM in synaptic vesicle priming and pre-synaptic VGCC organization (Jorgensen and Mango, 2002; Oh et al., 2021). To probe the role of RIM-dependent LLPS in motor outputs, we utilized a combination of small molecule and genetic manipulations.

Utilizing two mutants, the hypomorphic allele, *unc-10 ( e102 )* , and a null mutation (i.e., complete loss-of-function), *unc-10 ( md1117 )* (Fig. 1a), we conducted swimming assays in late stage (L4) larvae under varying treatment conditions (Fig. 1b). Both *unc-10* mutants show robustly disordered locomotion, with a decreased average swimming speed (Fig. 1d), increased curling behavior (Fig. 1g), and a decreased wave initiation rate (Fig. 1j) relative to wild-type (WT) controls (Extended Data 1-3). To determine if RIM liquid condensates contribute to these behaviors, we used aliphatic alcohols to disrupt liquid complexes independent of solid like assemblies (Kroschwald et al., 2017). The aliphatic alcohol, 1,6-hexanediol (1,6-HD), specifically disrupts hydrophobic phase separation mediated interactions and has recently been used in neurons to melt liquid assemblies in real time (Berkeley and Debelouchina, 2022; Park et al., 2023; Guzikowski and Kavalali, 2024). Therefore, we conducted swimming assays of the three different genotypes in 1,6-HD in combination with negative controls 2,5-hexanediol (2,5-HD) and glycerol.

With 1% 2,5-HD, 1% 1,6-HD, and 3% glycerol treatment, we did not detect a significant effect on swimming speed (Fig 1e) or wave initiation rate (Fig 1k), but 3% glycerol results in an increased prevalence of a stationary posture or “curling” (Fig. 1h, Extended Data 4) (Ezcurra et al., 2011). Treatment of WT animals with 3% 2,5-HD and 3% 1,6-HD (equivalent to concentrations used in *in vitro* studies) resulted in decreased swimming speed (Fig. 1f) and wave initiation rate (Fig. 1l), with marginal changes in curling (Fig. 1i). Remarkably, treatment of the UNC-10 mutants with 3% aliphatic alcohols did not result in additional impairment of their swimming locomotion (Fig. 1f, i, l Extended Data 5-8). While we cannot exclude a floor effect on swimming locomotion in the UNC-10 mutants, the inhibitory effect of aliphatic alcohols appears to require RIM function. Interestingly, we did not observe a significant difference between 3% 1,6-HD and the negative control 3% 2,5-HD treatment, suggesting a potential absence of functional specificity between these two alcohols in *C. elegans* at the concentrations used (Lin et al., 2016; Düster et al., 2021). Future work investigating the specificity and utility of different aliphatic alcohols to probe LLPS in different model systems is necessary to fully utilize these small molecules as a tool to investigate synaptic physiology. In summary, treatment with aliphatic alcohols disrupted swimming movements in WT worms but this effect was occluded in UNC-10 mutants, suggesting that UNC-10-dependent LLPS could contribute to these aspects of motor behavior.

How the dynamics and utility of pre-synaptic LLPS shift throughout development and maturation is still largely unclear. Previously, in *C. elegans ,* LLPS has been shown to be instrumental in active zone formation, in which Liprin-α and ELKS-1 condensation mediates active zone assembly, upstream of RIM/ UNC-10 organization. Interestingly this liquid-like active zone assembly transitions to a hydrogel with maturation (McDonald et al., 2020; McDonald and Shen, 2021). Importantly, in several studies LLPS was probed via FRAP (Fluorescence Recovery After Photobleaching) of relatively large microscale regions (Tsuriel et al., 2009; McDonald et al., 2020; Emperador-Melero et al., 2021), although increasing evidence suggests that LLPS operates within the nanoscale of a given synapse to regulate function (Guzikowski and Kavalali, 2024; Zhang et al., 2025). Since liquid assemblies exist on a spectrum of biophysical states with different stabilities, dynamics and viscosities, the scale at which LLPS operates may also be developmentally regulated. For example, during development, LLPS could operate at the micrometer scale to drive synaptic assembly and then, later, function at nano-scale to drive synaptic signaling in mature neurons, specifically within the synaptic active zone nanocluster. Importantly, phase separation is suggested as a mechanism underlying neurodegenerative diseases characterized by aggregate formation (Zbinden et al., 2020). However, the determinants of the equilibrium of physiological versus pathological LLPS during ageing remain unknown. The disentanglement of the temporal dynamics of LLPS is paramount to elucidating the functional significance of synaptic liquid assemblies.

Beyond investigating a behavioral *in vivo* correlate to pre-synaptic LLPS studies, these data suggest that aliphatic alcohols can be used as a tool to conduct genetic screens. *In vitro* isolated liquid condensates grow robustly, with a two-protein condensate reaching a size many-fold larger than an entire synapse. However liquid condensates function at nano-scale within a synapse (Guzikowski and Kavalali, 2024), raising the question of how the sizes of liquid condensates are controlled. *C. elegans* offer an easily manipulatable genetic system amenable to pharmacological treatment, providing an especially useful organism for elucidating the molecular mechanism that defines the dimensions of liquid condensates. Novel approaches to specifically manipulate LLPS mediated complexes – independent of targeted structured protein domain interactions – are necessary to establish the nano-specificity and physiological relevance of LLPS. In this nascent field, the use of *C. elegans* as a model organism will be advantageous in the association of distinct LLPS motifs with neurotransmission and behavior.

## Methods

*Strains and genetics*

Nematode strains were maintained at 23°C with standard culture techniques (Brenner, 1974). Only hermaphrodite animals were used. All animals were late larval 4 (L4) stage for experiments.

**Table:** 

Strain	Genotype	Available from
N2	WT	Caenorhabditis Genetics Center
CB102	*unc-10 ( e102 ) *	Caenorhabditis Genetics Center
NM1657	*unc-10 ( md1117 ) *	Caenorhabditis Genetics Center

*Swimming Assay*

Drug treatments used were 1,6-hexanediol (Sigma Aldrich 240117), 2,5-hexanediol (Sigma Aldrich H11904), and glycerol (Sigma Aldrich G2025) at 1% and 3% mass to volume ratio in M9 buffer. Control group was M9 buffer (3 g KH _2_ PO _4_ , 6 g Na _2_ HPO _4_ , 5 g NaCl, 1 ml 1 M MgSO _4_ , H _2_ O to 1 liter). Treatment length was one-minute with swimming assay conducted in treated buffer. Briefly, animals were moved from agar plate to a glass dish with 40 µl of buffer, following one-minute treatment exposure, swimming was recorded using WormLab 2023.1.1 (MBF Bioscience LLC, Williston, VT USA). Each swimming assay was conducted on individual worms. The WormLab software was used to record videos, conduct locomotion tracking, and data analysis.

The three metrics assessed were swimming speed (traveling speed of the animal over a two-stroke interval), the curling behavior (percentage of time the animal spent bent around and overlapped with themself), and wave initiation rate (the number of body waves initiated from the head or tail per minute).

*Statistical Analysis*

A Shapiro-Wilk test for normality was run to select the appropriate parametric or nonparametric statistical analysis. When comparing three or more groups a one-way ANOVA or nonparametric Kruskal-Wallis with Dunnett's or Dunn's post hoc multiple comparisons tests were conducted as appropriate. Each group was compared within its own genotype. p< 0.05 was considered statistically significant. Data presented as mean ± standard error of mean (SEM).

## Data Availability

Description: Extended Data-1. Resource Type: Audiovisual. DOI: https://doi.org/10.22002/59y0y-m4458 Description: Extended Data-2. Resource Type: Audiovisual. DOI: https://doi.org/10.22002/z2f3x-4ah90 Description: Extended Data-3. Resource Type: Audiovisual. DOI: https://doi.org/10.22002/rna1w-xt058 Description: Extended Data-4. Resource Type: Audiovisual. DOI: https://doi.org/10.22002/y3wa3-n4d26 Description: Extended Data-5. Resource Type: Audiovisual. DOI: https://doi.org/10.22002/gm48e-6t470 Description: Extended Data-6. Resource Type: Audiovisual. DOI: https://doi.org/10.22002/4pkvr-ar484 Description: Extended Data-7. Resource Type: Audiovisual. DOI: https://doi.org/10.22002/fzrz8-dmd73 Description: Extended Data-8. Resource Type: Audiovisual. DOI: https://doi.org/10.22002/s8dz9-45e34
